# Invest in Primary Health Care and Public Health for the Pandemic and Beyond: An ASPHER Statement

**DOI:** 10.3389/phrs.2022.1604875

**Published:** 2022-05-30

**Authors:** Henrique Lopes, Alison McCallum, Jose M. Martin-Moreno, John Middleton

**Affiliations:** ^1^ Public Health Unit, Health Sciences Institute, Catholic University of Portugal, Lisboa, Portugal; ^2^ Centre for Population Health Sciences, Usher Institute, University of Edinburgh, Edinburgh, United Kingdom; ^3^ Department of Preventive Medicine and Public Health, Medical School and INCLIVA, University of Valencia, Valencia, Spain; ^4^ Wolverhampton University, Wolverhampton, United Kingdom; ^5^ Association of Schools of Public Health in the European Region (ASPHER), Brussels, Belgium

**Keywords:** public health, pandemic, primary health care, COVID-19, ASPHER

## Shifting Pattern of Health Care Response to Pandemic Phases

Governments and health authorities must invest more in primary health care (PHC). This is increasingly urgent to assure service continuity throughout and beyond the pandemic [1, 2]. The pandemic hit hospitals hardest and most visibly in the unprepared health systems of European countries [3, 4]. PHC committed early to new models of working, enforced by lockdowns, accelerating the digital revolution [1].

## A Strong Primary Health Care Role in Mass Vaccination

Mass vaccination in 2021 demanded proactive involvement across sectors, especially PHC, public health (PH), community services and the third sector [2]. Inequalities in vaccine uptake could only be addressed and overcome by community organisations sensitive to local needs and beliefs, alongside trusted PHC professionals. The recent Omicron wave has been characterised by severe service disruption in key areas of the economy, through sickness absence and uncontrolled infection in children.

## The Need for the “Vaccines-Plus”/“Do It All” Approach?

Governments have not followed a “Vaccine-plus” approach failing to use all preventive measures available [5]. Mandates were removed, replaced by appeals to individual behaviours. Unsurprisingly, infection rates have not declined as hoped, creating new burdens for PHC. The impacts of long-COVID, mental exhaustion and “pandemic fatigue” are clear [6]. Virtually all care occurs in the community and additional gaps in PHC, school nursing, occupational and mental health are evident. There has never been a clearer need to invest in and plan for better integration of primary and community health care, complementing an expanded, resourced and trained multidisciplinary PH workforce [7].

## Omicron Requires Strengthened Primary and Community Services

The avalanche of patients with less severe COVID-19 is shifting care progressively from hospitals to PHC, but without plans in place to strengthen investment and reallocate staffing, as happened in hospitals during earlier waves. As society reopens while case rates remain high, PHC and community services remain fragile [8]. The Delta and Omicron waves saw increasing exposure of children and younger adults. PHC provides upwards of 90% of health care and is the main source of assessment and support for people with multimorbidity. PHC has the potential to address health inequalities.

## Failing to Invest in Primary Health Care-Learning From History?

It is essential to learn from experiences in countries such as Canada after the shock of SARS in 2003, which identified the need for better coordination and integration of PH services with health care structures, particularly PHC. PHC services have also recognised opportunities for permanent and beneficial change brought about by the pandemic, especially digital technologies [9].

## Unsustainable and Unequal Pandemic Health Care

As we strengthen PHC, public and community health, we must build more sustainable, approaches, increasing protection of our local and global eco-system and environment, following the “One Health” philosophy [10]. We have failed to deliver vaccine equity [11].

## An ASPHER Statement

ASPHER believes that health administrations, local and national governments should:

(1) Understand and act on the principle that preparedness and response begins with communities, PH, and PHC services.
(2) Assume a multi-disciplinary approach to creating and disseminating new knowledge and understanding, responding to challenges posed by the pandemic: prevention, awareness, diagnosis, treatment, recovery. Health and science literacy, modern tools and communication must be within reach of all.
(3) Ensure that the core COVID-19 approach is coordinated by pivoting investment towards PHC, interconnected with PH services, clarifying this does not mean hospitals withdraw from providing needed care.
(4) Sustainably strengthen PHC with necessary human, financial and technological means, with investment levels reflecting community need.
(5) Invest in universal community and homecare services, meeting need and reducing hospital and PHC centre presentations.
(6) Address the need to support children’s health in the community, including school nursing and assure health services complement PHC.
(7) Review availability of occupational health services, seeking to grow these to meet workforce health care needs.
(8) Invest in community development and voluntary services to support preventive efforts tackling inequalities, support vulnerable people and address needs caused by the pandemic
(9) Create funded plans for strengthening PHC, including mental health care and community services, enhancing support for people with chronic conditions, ensuring better longitudinal and coordinated care and minimising the risk of unconnected or non-integrated hospital services and managing increases in multimorbidity arising as a direct consequence of long COVID.
(10) Build sustainable, less wasteful approaches giving attention to animal health, eco-system and environment protection, following the “One Health” approach.

### Conclusion

The end of the pandemic is not determined by the pronouncements of politicians or journalists. We will not be free of the virus, until everyone is free [12]. All interventions we have against the virus still need to be applied. Careless relaxations of PH measures in rich countries, inadequate support for local PH and PHC and the communities they serve, and failure to support the global delivery of vaccines keeps us all in a state of perpetual COVID*.* We must invest in local PH and PHC and communities to extract us from this pandemic and provide better health for the future.

## Author’s Note

The Association of Schools of Public Health in the European Region (ASPHER) is the key independent European organisation dedicated to improving and protecting the public health by strengthening education and training of public health professionals for both practice and research. www.aspher.org.

## Society Note

ASPHER is responsible and liable for all contents of ASPHER statements. ASPHER statements are approved by an Editor-in-Chief but not externally peer reviewed.
